# Cash transfers to enhance TB control: lessons from the HIV response

**DOI:** 10.1186/s12889-018-5962-z

**Published:** 2018-08-22

**Authors:** William E. Rudgard, Daniel J. Carter, James Scuffell, Lucie D. Cluver, Nicole Fraser-Hurt, Delia Boccia

**Affiliations:** 1 Department of Infectious Disease Epidemiology, London School of Hygiene & Tropical Medicine, Keppel Street, London, WC1E 7HT UK; 2 Epsom and St Helier University Hospitals NHS Trust, London, UK; 3 Department of Social Policy & Intervention, University of Oxford, Oxford, UK; 4 Department of Psychiatry and Mental Health, University of Cape Town, Cape Town, South Africa; 5 World Bank Group, Washington, DC USA

**Keywords:** Tuberculosis, HIV, Social determinants, Prevention, Social protection, And cash transfer

## Abstract

**Background:**

The World Health Organization prioritises a more holistic global response to end the tuberculosis (TB) epidemic by 2030. Based on experiences in the HIV response, social protection, and in particular cash transfers, show promise for contributing to this. Currently, individual-level evidence for the potential of cash transfers to prevent TB by addressing the structural social determinants of disease is lacking. To identify priority actions for the TB research agenda, we appraised efforts by the HIV response to establish the role of cash transfers in preventing HIV infection.

**Main body:**

The HIV response has evaluated the effects of cash transfers on risky sexual behaviours and HIV incidence. Work has also evaluated the added effects of supplementing cash transfers with psychosocial support. The HIV response has focused research on populations with disproportionate HIV risk, and used a mix of explanatory evaluations, which use ideal conditions, and pragmatic evaluations, which use operational conditions, to generate evidence that is both causally valid and applicable to the real world. It has always collaborated with multiple stakeholders in funding and evaluating projects. Learning from the HIV response, priority actions for the TB response should be to investigate the effect of cash transfers on intermediary social determinants of active TB disease, and TB incidence, as well as the added effects of supplementing cash transfers with psychosocial support. Work should be focused on key groups in high burden settings, and look to build a combination of explanatory and pragmatic evidence to inform policy decisions in this field. To achieve this, there is an urgent need to facilitate collaborations between groups interested in evaluating the impact of cash transfers on TB risk.

**Conclusions:**

The HIV response highlights several priority actions necessary for the TB response to establish the potential of cash transfers to prevent TB by addresing the structural social determinants of disease.

## Background

In 2016, an estimated 10.4 million people developed active tuberculosis (TB) disease, and it was the leading infectious killer in the world [1]. The Sustainable Development Goals (SDGs) have called for the global TB epidemic to be ended by 2030 [2]. The indicator chosen to monitor progress towards this target is TB incidence. At approximately 2%, the current annual decline in global TB incidence is far below the 10% that is projected to be necessary for achieving the SDG’s ambitious target [1]. Historically, rapid annual declines in TB incidence have only been achieved in the context of universal health coverage combined with broader social and economic development [3]. Recognising this, the World Health Organization has called for a more holistic TB response that includes social protection, poverty alleviation, and action on the social determinants of disease [4]. Model projections show that increased focus on these activities could have a significant impact on global TB rates [5].

Social protection is defined as actions to help families prevent, mitigate or cope with risks that may lead to, or exacerbate poverty and deprivation [6]. Cash transfer initiatives are a form of social protection that provide regular and predictable small amounts of money to eligible poor families [7]. Cash transfers may be given unconditionally, or conditional on household members’ participation in education, health and nutrition services [7]. Both unconditional and conditional cash transfer initiatives aim to increase poor families’ consumption potential. However, by making receipt of cash transfers contingent on utilization of public services, conditional initiatives also encourage recipients to invest in knowledge, skills, and abilities [8]. Cash transfers have been endorsed as a key component of the World Health Organization’s End TB strategy [4]. However, the body of evidence informing their potential contribution to eliminating TB remains limited [9].

A major stimulus for the endorsement of cash transfers in the TB response has been evidence of their ability to address structural social determinants of human immunodeficiency virus (HIV) infection, and enable access to critical biomedical HIV services (e.g., voluntary counselling and testing, and HIV treatment) [10]. Since then, the TB response has begun to evaluate the impact of cash transfers on equivalent indicators relevant for TB control. One systematic review shows how poverty-reduction cash transfers positively impact on income poverty and food availability as structural and intermediary social determinants of active TB disease, respectively, and one ecological study in Brazil, shows an association between higher municipal coverage of cash transfers and lower TB incidence [11, 12]. Alternatively, individual-level evidence in Brazil, Moldova, and Peru, demonstrates the potential of cash transfers to enable access to TB treatment, measured by a reduction in peoples’ risk of adverse TB treatment outcomes including abandoning or failing treatment, or dying during treatment [13–16]. An ongoing community randomised control trial in Peru is also evaluating the use of cash transfers in this way [17, 18].

Substantial reductions in annual TB incidence, like those necessary to end the TB epidemic by 2030, will only be achieved via a combined approach that both prevents development of active TB disease and supports successful treatment [19]. Preliminary evidence suggests that cash transfers have the potential to support both of these activities. Currently, important individual-level evidence is still lacking to inform their potential to prevent TB via action on the structural social determinants of disease. In comparison, the HIV response has invested greatly in establishing the potential of cash transfers to prevent HIV infection in this way. With the aim of identifying priority actions for establishing the role of cash transfers in preventing TB, we appraised efforts by the HIV response to establish the role of cash transfers in preventing HIV infection.

## Main body

### Summary of past actions by the HIV response to establish the role of cash transfers in HIV prevention

**Fig. 1:**
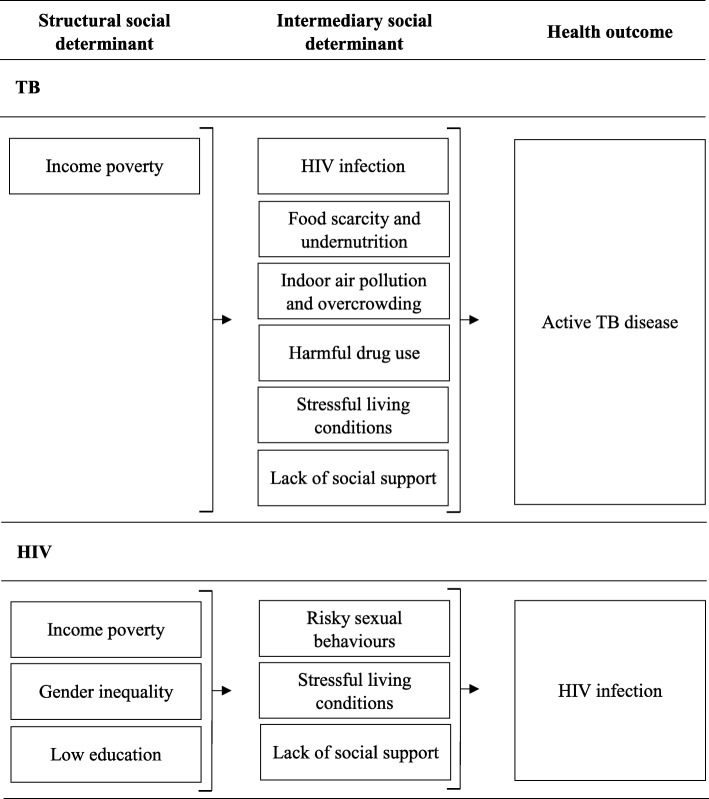
Framework of selected structural and intermediary social determinants of active TB disease, and HIV infection. Only structural and intermediary social determinants of health mentioned in the text are included in the framework. Structural social determinants of health include components of peoples’ socioeconomic position that indirectly influence health outcomes by acting on intermediary social determinants of health [62]. Intermediary social determinants of health include material circumstances, behaviours and biological factors, and psychosocial factors that directly influence health outcomes [62]

Predominantly sexually transmitted, HIV is inextricably linked with high-risk sexual behaviours that are at least partially shaped by underlying structural social determinants of health including income poverty, gender inequality, and low education (Fig. 1) [20]. In the HIV response, interest in cash transfers grew from an understanding of their potential to mitigate income poverty, increase children’s school attendance, and boost family health-seeking behaviours [21]. Since then, the HIV response has sought to support cash transfer programme implementation by generating evidence for their effects on high-risk sexual behaviours, and HIV prevalence and incidence.

With more than 70% of the global burden of HIV concentrated in sub-Saharan Africa, most of the work on cash transfers in the HIV response has been conducted there [22]. The primary target populations for research have been impoverished orphans and vulnerable children (OVC), and adolescent girls and young women, which both experience disproportionately high rates of HIV infection [22]. Research has been limited in other key populations (e.g., migrant communities, sex workers, and injecting drug users) [10]. Progress in generating evidence for the impact of cash transfers on HIV risk has relied on strong collaborations between international, governmental, non-governmental, and civil-society organisations across a range of sectors [23]. This approach has built on a paradigm that arose early on in the HIV response, whereby limited availability of biomedical interventions encouraged greater focus on disease prevention via behavioural interventions [24]. To ensure both causal validity and real-world applicability of findings, evidence has been generated from both explanatory and pragmatic evaluations [25, 26]. Work has also evaluated the effect of cash transfers provided conditional on school attendance, and separately in combination with psychosocial support.

To provide evidence and context for these actions, we summarise three relevant examples of efforts by the HIV response to establish the role of cash transfers in HIV prevention.

#### Cash transfers for orphans and vulnerable children in Kenya

The first evaluation of cash transfers in the HIV response started in Kenya in 2003. The process began with an unsuccessful funding application to the Global Fund for HIV, TB and Malaria [23]. Following this, the Ministry of Home Affairs, joined together with The United Nations Children’s Fund (UNICEF), the Swedish International Development Agency (SIDA), the Norwegian National Committee for UNICEF, and local communities to evaluate the impact of a pilot unconditional cash transfer initiative for orphans and vulnerable children (CT-OVC) on structural and intermediary social determinants of HIV [23, 27]. Spread across multiple phases (pre-pilot, pilot, and expansion), the evaluation evolved from a pre-post analysis to a cluster randomised control trial with long-term follow-up [23]. Results from the initial pre-post analysis were instrumental for convincing large funders, like the World Bank, of the value of investing in cash transfer initiatives, and therefore securing the means to conduct more complex evaluations [23]. In 2011, results from the randomised evaluation showed receipt of unconditional cash transfers from the CT-OVC to be associated with reduced risk of early sexual debut [28]. As a pragmatic randomised control trial, results were both causally valid and applicable to the real world. This evidence helped secure domestic buy-in of the CT-OVC programme, which now supports over 250,000 households nationwide [23].

#### Cash transfers to encourage school attendance in Malawi and South Africa

Work in Malawi and South Africa has focused on establishing the potential of cash transfers provided to adolescent girls and young women (aged 15 to 24) conditional on their school attendance to reduce HIV risk [29, 30]. This approach intends to empower adolescent girls and young women’s decision making around sex. Evaluations have been conducted under ideal trial conditions, and therefore provide a reliable indicator for the efficacy of the intervention, but might not be generalizable to real world settings. In Malawi, receipt of conditional cash transfers over 18 months was found to significantly reduce age-disparate sex, and HIV prevalence [29]. Importantly, this effect was observed regardless of whether cash transfers were given unconditionally or conditional on study participants’ school attendance. Alternatively, in South Africa, receipt of conditional cash transfers over 22 months significantly reduced reports of recent unprotected sex (i.e., in the three months prior to interview), but did not reduce HIV incidence (considered to be a more accurate measure than HIV prevalence of the state of the epidemic) [30]. A potential source of bias in this study was that baseline receipt of a local governmental cash transfer initiative was high in both trial arms (≈80%). Furthermore, because the study eligibility criteria excluded young girls not attending school, the study was unable to estimate the potential of conditional cash transfers to reduce HIV incidence in this key vulnerable group [31].

#### Cash transfers and supplementary psychosocial support in South Africa

Recent efforts have evaluated the impact of governmental child-focused cash transfer initiatives on high-risk sexual behaviours in adolescents in South Africa [32]. Because the governmental initiatives were already implemented nationally, the evaluation could not use a randomised design. Instead it used a quasi-experimental design that simulated randomisation by matching treatment and control groups for variables that predicted their likelihood of receiving cash transfers [32]. Results showed that receipt of unconditional cash transfers from governmental child-focused programmes are associated with reduced risk of age-disparate sex and transactional sex [32]. Building on this evidence, work in South Africa has also explored a “cash plus care” model of delivering cash transfers, which combines economic support from cash transfers with psychosocial support from counselling and/or positive parenting [33]. The approach aims to mitigate income poverty, and simultaneously help people cope with a history of stressful living conditions and lack of social support. Still focusing on adolescents, this work is targeted at particularly high-risk groups within this population (e.g., people living with acquired immune deficiency syndrome (AIDS), and/or in informal housing). In South Africa, “cash plus care” has been shown to more effectively reduce the likelihood of adolescent girls and boys engaging in risky sexual behaviours compared to cash transfers alone [33]. Based on these results, in 2016, the Global Fund for HIV, TB and Malaria awarded South Africa US$ 50 million to provide “cash plus care” to over 40,000 girls nationally. The approach has also been identified as one of the evidence-based interventions of the Determined, Resilient, Empowered, AIDS-free, Mentored, and Safe (DREAMS) partnership, which is implemented in 10 countries across Southern and Eastern Africa, and reaches 1.8 million people [34].

### Summary of priority actions for the TB response to establish the role of cash transfers in TB prevention

Similar to HIV, TB is inextricably linked with several intermediary social determinants of health which are all largely shaped by income poverty (Fig. 1) [35]. Learning from the HIV response, evaluating the effect of cash transfers on these intermediary social determinants of TB risk is a key step in establishing the potential of cash transfers to prevent TB. The intermediary social determinants estimated to contribute most to the global TB burden are HIV, food scarcity and undernutrition, tobacco use, and indoor air pollution [3]. Evidence already exists for the effect of cash transfers on some of these factors. According to work by the HIV response, cash transfers may reduce HIV prevalence [29]. There is also evidence in Ghana, Kenya, Malawi, Mexico and Zambia for the effects of cash transfers on various indicators of food scarcity and undernutrition [36–38]. Results suggest that program design and operational performance are key for achieving significant impacts on these outcomes [37]. They also indicate that the sustainability of impacts is closely linked to macroeconomic factors such as inflation [37]. In Africa, Asia, and Latin America cash transfers have been shown to have no significant effect on tobacco use [39]. Where evidence is currently lacking, is for the effect of cash transfers on indoor air pollution. By reducing poverty, cash transfers might enable people to use cleaner modern fuels compared to polluting solid fuels like wood or charcoal [40]. In Brazil and Ecuador, cash transfers have been used to subsidise modern cooking fuels [41, 42], and there are ongoing discussions for using this approach in India too [43]. Where new initiatives are ongoing, efforts should be made to evaluate their impact on a measurable indicator of indoor air pollution.

The intended outcome of modifying intermediary determinants of TB risk is to prevent active TB disease. Therefore, as in the HIV response, it will also be necessary to generate individual-level evidence for the effect of cash transfers on disease incidence. This is likely to be especially important for the TB response, as unlike HIV infection and high risk sexual behaviours, developing active TB disease is not as strongly linked to a definite risk factor. Furthermore, previous work has identified a number of challenges with translating the measured impact of cash transfers on intermediary social determinants of TB into a potential impact on TB incidence [44].

Similar to how research on cash transfers in the HIV response has been focused in sub-Saharan Africa, which concentrates 70% of the global burden of HIV, studies investigating the effects of cash transfers on TB risk would have greatest value in the 30 countries that concentrate 89% of the global burden of TB [1]. It will also be important for this work to be focused on specific populations with disproportionate risk of TB. One such population is young adults (aged 20 to 35), especially in sub-Saharan African countries where TB risk is closely linked to the HIV epidemic [45]. Another, is people living in urban poor settlements with high population densities and crowded living conditions [45–49]. Commonly these are rural-to-urban migrants who also experience high-levels of depression and anxiety from factors including economic pressures, high work load, family separation, and discrimination [50–52]. Older people, drug users, and ethnic minorities are three other key populations with disproportionate risk of developing active TB disease [53–57].

According to the HIV response, establishing the impact of cash transfers on intermediary social determinants of TB and TB incidence will rely heavily on cross-sectoral collaborations between diverse stakeholders. As things stand, readily available microbiological diagnosis, anti-TB drugs, and the Bacillus Calmette-Guérin (BCG) vaccine, promote a mostly biomedical TB prevention paradigm. Efforts are therefore required to facilitate strong partnerships between stakeholders interested in evaluating the impact of cash transfers on the social determinants of active TB disease. Similar to that which was carried out for the CT-OVC in Kenya, a priority is to generate a minimum level of evidence that can be used to convince large funders of the value of investing in cash transfers for preventing TB. One platform aiming to achieve this is the Health and Social Protection Action Research & Knowledge Sharing (SPARKS) network, which brings together researchers, policy makers and implementers, Ministry of Health representatives, staff from United Nations organisations, civil society representatives and funding agencies interested in social protection and TB control [58]. Experiences by the HIV response demonstrate the relative speed at which a body of evidence can lead to large-scale roll-out of initiatives.

In the process of establishing the impact of cash transfers on TB risk, learning from the HIV response, it will be important to generate a mix of evidence from both explanatory and pragmatic evaluations. Explanatory evaluations can test the efficacy of an intervention, and compare different implementation designs such as unconditional versus conditional cash transfers. Learning from the HIV response, it will be important for any such evaluation to consider contextual factors of study settings like existing receipt of governmental cash transfers by households, and ensure that key vulnerable populations are included in study populations. Pragmatic evaluations can inform whether an intervention works in the real world. Given the increasing number of countries around the world with ongoing governmental cash transfer initiatives, this type of evaluation is ever-more possible [7]. For these initiatives, whilst it might be too late to conduct randomised pragmatic evaluations, like has been used in the HIV response, quasi-experimental methods can be an effective way to evaluate their impact on disease risk [32]. Several documents are available to guide quasi-experimental research designs [59]. Wherever possible, projects should aim to address the maximum number of evidence gaps for the effects of cash transfers on peoples’ risk of active TB disease.

In direct comparison to the HIV response, evaluations of cash transfers and TB risk should also consider the potential added impacts of a “cash plus care” model that combines cash transfers with psychosocial support. Similar to the HIV response, this approach might be especially relevant for high-risk population sub-groups [60]. Cash transfers together with psychosocial support (e.g., counselling, vocational training) might help relieve rural-to-urban migrant families’ economic pressures, and find solutions to their stressful living conditions. A similar approach might also be effective amongst drug users who are known to experience multiple overlapping risk factors including food scarcity, overcrowding, stressful living conditions, and high levels of comorbidity. In this specific population, “cash plus care” would likely benefit from including harm reduction strategies such as supervised drug consumption, and opioid replacement therapy [61].

## Conclusion

Meeting the ambitious goals of the SDGs will require simultaneous action to prevent development of active TB disease, and support successful TB treatment completion. Cash transfers might contribute to this. Whilst evidence is accumulating for their potential to support TB treatment completion, evidence informing their potential to prevent TB by addressing the structural social determinants of disease remains limited. Several priority actions for strengthening this body of evidence can be identified from previous efforts to establish the role of cash transfers in the HIV response. Work is now needed by the TB response to mobilise these actions.

## Abbreviations

AIDS: Acquired immune deficiency syndrome
BCG: Bacillus Calmette-Guérin
CT-OVC: Cash transfer for orphans and vulnerable children
DREAMS: Determined, Resilient, Empowered, AIDS-free, Mentored, and Safe
HIV: Human immunodeficiency virus
OVC: Orphans and vulnerable children
SDG: Sustainable Development Goals
SIDA: Swedish International Development Agency
SPARKS: Health and Social Protection Action Research & Knowledge Sharing
TB: Tuberculosis
UNICEF: The United Nations Children’s Fund
